# Does Pain Extent Predict Ongoing Pain and Disability in Patients with Chronic Whiplash-Associated Disorders?

**DOI:** 10.3390/jcm11030555

**Published:** 2022-01-22

**Authors:** Ahmed Alalawi, David W. Evans, Bernard Liew, Anneli Peolsson, Nicola Heneghan, Alison Rushton, Gunnel Peterson, Marco Barbero, Deborah Falla

**Affiliations:** 1 Centre of Precision Rehabilitation for Spinal Pain (CPR Spine), School of Sport, Exercise and Rehabilitation Sciences, University of Birmingham, Birmingham B15 1JN, UK; amalawi@uqu.edu.sa (A.A.); d.w.evans@bham.ac.uk (D.W.E.); N.Heneghan@bham.ac.uk (N.H.); A.B.Rushton@bham.ac.uk (A.R.); 2 Physical Therapy Department, College of Applied Medical Sciences, Umm Al-Qura University, Makkah 24382, Saudi Arabia; 3 School of Sport, Rehabilitation and Exercise Sciences, Faculty of Physiotherapy, University of Essex, Colchester CO4 3WA, UK; bl19622@essex.ac.uk; 4 Department of Medical and Health Sciences, Linköping University, SE-581 83 Linköping, Sweden; anneli.peolsson@liu.se (A.P.); gunnel.peterson@liu.se (G.P.); 5 Occupational and Environmental Medicine Center, Department of Health, Medicine and Caring Sciences, Unit of Clinical Medicine, Linköping University, SE-581 83 Linköping, Sweden; 6 Centre for Clinical Research Sörmland, Uppsala University, SE-751 05 Uppsala, Sweden; 7 Rehabilitation Research Laboratory, Department of Business Economics, Health and Social Care, University of Applied Sciences and Arts of Southern Switzerland, 6928 Manno, Switzerland; Marco.Barbero@supsi.ch

**Keywords:** whiplash injury, outcome, widespread pain, pain drawings, prognosis

## Abstract

This study investigates whether baseline pain extent, extracted from an electronic pain drawing, is an independent predictive factor of pain and disability measured 1 year and 2 years later in people with chronic WAD. Participants completed questionnaires assessing neck pain intensity, disability via the Neck Disability Index (NDI), psychological features, and work ability. Participants also completed electronic pain drawings from which their pain extent was extracted. A two-step modelling approach was undertaken to identify the crude and adjusted association between pain extent and NDI measured at 1-year and 2-year follow-ups. A total of 205 participants were included in the analysis. The univariate analysis showed that pain extent was significantly associated with the NDI score at the 1-year (*p* = 0.006, 95% CI: 0.159–0.909) and 2-year (*p* = 0.029, 0.057–0.914) follow-ups. These associations were not maintained when we introduced perceived disability, psychological health, and work ability into the model after 1 year (*p* = 0.56, 95%CI: −0.28–0.499) and 2 years (*p* = 0.401, −0.226–0.544). Pain extent, as an independent factor, was significantly associated with perceived pain and disability in patients with chronic WAD for up to 2 years. This association was masked by neck disability, psychological health, and work ability.

## 1. Introduction

The primary cause of whiplash-associated disorders (WAD) is motor vehicle collision, which results in a neck injury due to the acceleration–deceleration mechanism [1]. In developed countries, the annual incidence of WAD is estimated to be >300 per 100,000 inhabitants [2], placing a significant annual socioeconomic burden of USD 13.4 billion and USD 3.9 billion on Europe and the USA, respectively [3,4]. Improvements in pain and disability are likely to occur in the first three months after the injury but do not change substantially after this time. Approximately 50% of patients with WAD continue to experience persistent symptoms one year after the trauma [5,6].

Prognosis following WAD is multifactorial and may include personal, social, and environmental factors. Indicators of central sensitization appear to influence recovery in people with WAD [7,8,9]. For example, widespread pain is often seen in patients with chronic WAD [10] and has been associated with higher pain and disability WAD [11]. Several studies have shown that people with chronic WAD have widespread hypersensitivity [12,13], including thermal hyperalgesia [14], mechanical hyperalgesia [14], and hyperexcitability of spinal cord reflexes [15,16]. Initial high scores of neck pain and disability have also been associated with persistent pain and disability in patients with WAD [17,18], as have psychological factors such as depression and anxiety [19,20,21], fear of movement [19], and lower self-efficacy [20]. Yet, the predictive ability of pain extent (i.e., the size of the painful area) on neck pain and disability has not been investigated in people with chronic WAD.

We hypothesized that individuals with chronic WAD who present with larger pain extent will report higher pain and disability in the longer term. Therefore, the aim of this study was to investigate whether pain extent, extracted from a pain drawing by individuals with chronic WAD, was an independent predictive factor of pain and disability when measured 1 year and 2 years later.

## 2. Materials and Methods

A secondary analysis of data from a randomized controlled trial (ClinicalTrials.gov, NCT01528579, 1 Febrary 2021) [22] was performed to investigate whether pain extent was associated with long-term outcome in people with chronic WAD. Pain extent was not extracted from the pain drawings and analyzed in the previously published clinical trial [22].

### 2.1. Participants

Participants were recruited to the original trial [22] from six different Swedish regions between February 2011 and May 2012. They were recruited from primary health care centers, orthopedic clinics, and hospital outpatient services. Participants were eligible if they were between 18 and 63 years of age and if the cause of the current symptoms was a whiplash injury occurring in the preceding 6 to 36 months. Additionally, the following criteria had to be met: classified as WAD grade II or III [1], at least 10/50 points on the Neck Disability Index (NDI) [23], and average pain intensity of more than 20/100 mm on a visual analogue scale (VAS) [24]. Participants were excluded if they had a previous neck injury with unresolved symptoms, neck surgery, traumatic brain injury, more than one month’s absence from work due to neck pain in the year preceding the injury, tumor, myelopathy, any other dominant pain complaint, or were not fluent in the Swedish language. The original trial investigated the effect of exercise interventions in individuals with chronic WAD with 3, 6, 12, and 24 months follow-up. For the original trial [22], participants were randomly allocated to one of three groups to receive: a neck-specific exercise intervention only (NSE); NSE, with the addition of a behavioral approach (NSEB); or prescribed physical activity (PPA).

The process of recruiting participants was as follows: 7950 potential participants were invited to participate by letter; of them, 7531 were excluded, as baseline self-reporting criteria were not fulfilled or refused to participate. A further 203 participants were excluded after eligibility assessment and physical examination, and the remaining 216 subjects were included in the trial. For further details, see the original trial report [22].

The Regional Ethics Committee of Linköping, Sweden approved the study. Written informed consent was obtained from all participants and the study was conducted according to the Declaration of Helsinki.

### 2.2. Outcome

Neck pain-related disability was the primary outcome, which was measured using the NDI at baseline and at 1 year and 2 years after the intervention. The NDI is a neck-specific questionnaire that consists of 10 items related to functional activities of daily life such as personal care, lifting, reading, work, driving, sleeping, and recreation [25]. Each item is scored from 0 (no disability) to 5 (complete disability) to give a maximum total score of 50, which can be expressed as a percentage (0–100%) with larger scores reflecting higher levels of disability. The NDI is a reliable (intraclass correlation coefficient up to 0.98) and valid measurement of disability in people with neck pain disorders [26].

### 2.3. Candidate Predictors

Participants completed self-reported questionnaires in order to collect baseline information about personal, demographic, psychological, and other related factors, as detailed below prior to randomization.

### 2.4. Pain Extent

Participants were asked to draw their perceived pain area on two body charts (frontal and dorsal view of the body). Pain drawings were then digitized and imported into custom-made image analysis software, developed with Matlab^®^. The reliability of this automated process of digitizing pain drawings has been established in people with chronic neck pain (intraclass correlation coefficient (2, 1) = 0.92) [27]. The software then quantifies the number of pixels shaded in the pain drawing (frontal and dorsal), expressed as a percentage of the total body chart area [11,27]. Further details are available elsewhere [11].

#### 2.4.1. Neck Pain Intensity

Current neck pain intensity was measured at baseline, utilizing the VAS where 0 = no pain and 100 = worst imaginable pain [24]. The validity and reliability of the VAS have previously been established [28].

#### 2.4.2. Pain Disability Index (PDI)

The PDI score of each participant was assessed to evaluate any aspects of their lives that were disrupted by pain. It is divided into seven categories, where each is given a score from 0 (no disability) to 10 (usual activities have been prevented by pain), producing a total score of seventy (greater disability due to pain). The validity and reliability of the PDI have been established previously [29].

#### 2.4.3. General Health

The EuroQol Five Dimension Scale (EQ-5D) was utilized to assess perceived quality of life. EQ-5D comprises five dimensions related to a patient’s mobility, self-care, usual activities, pain or discomfort, and anxiety or depression [30]. The EuroQol VAS provides a self-estimation of current health (0 to 100 points scale, with higher scores representing the best imaginable health state).

#### 2.4.4. Psychosocial Features

The Pain Self-Efficacy Scale (PSES) evaluated each participant’s confidence in managing activities despite pain. Participants scored 20 items relating to physical and psychological factors, rating each item 0–10. The final score ranged between 0–200, with highest scores indicating greater confidence [31]. The Pain Catastrophizing Scale (PCS) was utilized to evaluate evidence of rumination, magnification, or frustration regarding the participants’ pain control [32]. PCS scores range 0 to 52, where a higher score indicates greater levels of negative pain-related thoughts. The 11-item Tampa Scale of Kinesiophobia (TSK) was used to evaluate fear of movement, with higher scores reflecting a greater fear of movement (score range 11–44) [33]. The Hospital Anxiety and Depression Scale (HADS) was utilized to evaluate depression and anxiety and consists of seven items for anxiety (HADS-A) and seven for depression (HADS-D), producing a score between 0 and 21, with higher scores associated with heightened levels of anxiety and depression [34].

#### 2.4.5. Work-Related Factors

To assess self-perceived work ability, the Work Ability Index (WAI) was used, which consists of seven items considering physical and mental work demands in conjunction with an individual’s health status [35]. The validity and reliability of the WAI as a measure for work disability was confirmed [36].

### 2.5. Statistical Analysis

Descriptive summary statistics were performed on the participant’s baseline characteristics, including general health, pain, disability, work factors, and psychological factors.

All analyses were performed using R software, version 3.4.2. The analyses included all 205 participants from inception. Missing data were handled using the Multiple Imputation using Chained Equations (MICE) method carried out using R software’s “mice” package [37], with five imputations and 10 iterations per imputation.

To identify the predictive value of pain extent on NDI outcomes after 1 year and 2 years, a two-step modelling approach was undertaken [38]. First, the number of predictor variables entering the second stage analysis was reduced using a least absolute shrinkage and selection operator (LASSO) regression. LASSO regression was performed on the predictor variables of baseline NDI, PDI, TSK, PCS, HADS-A, HADS-D, EQ-5D, EQ-VAS, PSES, WAI, and VAS on the endpoint NDI across the five imputed datasets using a previously published method [38]. Second, two simple least-squared regressions were performed. The first regression was performed with only pain extent as the predictor, and the second regression was performed with pain extent, group, and all remaining predictors identified from the LASSO regression [39]. The first analysis provided a crude association of pain extent with NDI, whilst the second analysis provided an adjusted association of pain extent with NDI. The least-squared regression was performed on all five imputed datasets independently, and the results were pooled using Rubin’s rule from across the five analyses [37,40]. A *p* value of <0.05 was considered statistically significant.

In this study, the current guidelines for estimating sample size required for creating prognostic models were employed [41]. Several criteria should be specified, including the expected R^2^ of the model, the mean outcome value together with the standard deviation of the mean in the target population, and the number of potential predictors [41]. These data were derived from a similar study including individuals with chronic WAD in which R^2^ is 0.56, with mean NDI scores of 15.57 and standard deviation of 14.1 after 2 to 3 years post-accident [8]. Finally, seven potential candidate predictors were selected to be included in this study at the 1-year follow-up with six predictors at 2 years. This resulted in a sample size of 241 and 240 participants after 1 and 2 years, respectively. Sample size calculations were handled using the pmsampsize package, carried out using R software [41].

## 3. Results

The data from 205 participants were included in the analysis of this study, following the multiple imputation process. Forty-five (22%) and ninety-one (44%) of the participants’ data were missing an NDI score at one and two years, respectively. The characteristics of the individuals, including their sex, age, and other baseline measures, are presented in Table 1. Further details on participant characteristics can be found in the report of the randomized controlled trial [22].

### 3.1. Predictor Variable Selection (i.e., Shrinking the Number of Predictors)

The baseline covariates with nonzero coefficients to the NDI outcome were NDI, HADS-D, PSES, and WAI at 1 year and NDI, TSK, and WAI at 2 years (Table 2 and Table 3).

### 3.2. Prediction of Outcome at One Year 

A one-percent increase in pain extent significantly increased NDI by 0.5 units (t = 2.88, *p* = 0.006, 95% CI: 0.159–0.909) (Table 4). When the relationship between pain extent and NDI was adjusted by the baseline effects of NDI, group allocation, HADS-D, PSES, and WAI, a one-percent increase in pain extent non-significantly increased NDI by 0.11 units (t = 0.586, *p* = 0.56, 95% CI: −0.28–0.499) (Table 5).

### 3.3. Prediction of Outcome at Two Years

A one-percent increase in pain extent significantly increased NDI by 0.49 units (t = 2.383, *p* = 0.029, 95% CI: 0.057–0.914) (Table 6). When the relationship between pain extent and NDI was adjusted by the baseline effects of NDI, group allocation, TSK, and WAI, a one-percent increase in pain extent non-significantly increased NDI by 0.16 units (t = 0.856, *p* = 0.401, 95%CI: −0.226–0.544) (Table 7).

## 4. Discussion

This is the first study to investigate whether the reported spatial extent of pain is a predictive factor of outcome in chronic WAD. The results suggest that patients with chronic WAD who present with more widespread pain are expected to have increased ongoing pain and disability at 1 year and 2 years, even after participating in an exercise program. Yet, this association was not maintained when we adjusted for other factors. This finding therefore only partially supports our assertion that patients with chronic WAD reporting widespread pain continue to demonstrate higher persistent pain and disability at least 2 years later.

Pain extent explained 5% and 4% of the variance in the NDI in this population at 1 year and 2 years, respectively. A one-percent increase in baseline pain extent predicted a significant increase in NDI by 0.5 and 0.49 at 1 year and 2 years, respectively. Thus, patients who presented with more widespread pain were more likely to have ongoing neck pain and disability at least two years later. This result is consistent with earlier research confirming a relationship between larger area of pain and higher neck disability in patients with chronic WAD [11] and patients with chronic neck pain [27]. These results are also supported by earlier findings that a 10-week rehabilitation program, including exercises, resulted in a 37% reduction in neck pain intensity in people with WAD and signs of mechanical hyperalgesia, whereas the response to the same intervention was only a 16% reduction in neck pain intensity in people with WAD with signs of widespread mechanical and cold hyperalgesia, suggesting an up-regulation of central nociception processing and/or a loss of descending inhibition [42].

Widespread pain is characteristic of central sensitization, a phenomenon thought to contribute to the maintenance of pain and disability in people with WAD [10]. To date, however, there has been very little investigation of the relationship between pain extent and direct measures of central sensitization. In one study investigating individuals with chronic knee arthritis, larger pain extent was significantly associated with lower-pressure pain thresholds measured both over the knee and at a remote site [43]. Thus, pain extent, extracted from pain drawings, may be clinically useful when investigating evidence of central sensitization, an aspect that ought to be considered when determining prognoses. Further studies are needed to extend this work to a WAD population.

Earlier research measuring pressure pain thresholds over cervical, elbow, and tibialis anterior muscle sites showed a significant association between pressure pain threshold and poorer outcomes on the NDI up to 2–3 years following treatments by a physiotherapist or a chiropractor after a whiplash injury [8]. Although moderate correlations between pressure pain thresholds and disability have been found in people with WAD when measured only at cervical spine sites [44,45], the results from a meta-analysis found that patients with chronic WAD show higher pressure pain sensitivity at multiple sites in the body [46], suggesting that augmented central processing is present in this population. Moreover, a systematic review by Williams et al. [47], which explored prognostic factors in patients with WAD, found moderate evidence supporting the theory that cold hyperalgesia predicts higher neck disability in people with WAD. Further studies are required to explore the use of pain extent as an indicator of central sensitization in the prediction of outcome following physical interventions in patients with chronic WAD.

### 4.1. Adjusted Pain Extent

Pain extent was not predictive of NDI once we adjusted for NDI, HADS-D, PSES, and WAI after 1 year and NDI, TSK, and WAI after 2 years. Introducing these factors into the model lowered the beta coefficient (β) of pain extent from 0.53 to 0.11 and 0.48 to 0.15 at one and two years, respectively, suggesting that part of the association between pain extent and disability is explained by other factors such as perceived disability or psychosocial factors. Additionally, this could indicate that these elements may have a potential confounding effect on pain extent, blurring its association with the NDI when introduced into the model [48]. For a factor to be considered as a potential confounder, it must have an association with both the exposure and outcome [49]. In our study, and based on the proposed properties [49], we hypothesized that NDI, HADS-D, TSK, PSES, and WAI should be associated with widespread pain, given that the association between them and the overall outcomes in WAD have been established in earlier studies [17,18,19,20,21].

There is evidence of associations between the presence of psychological factors and widespread pain in patients with WAD. A study by Holm et al. [50] aimed to investigate whether psychological features and other injury-related factors were associated with the development of widespread pain, measured by the number of painful body areas, in patients with WAD; depressive mood was strongly associated with widespread pain, compared to those who present with localized pain (adjusted OR = 3.2). Similarly, in patients with chronic WAD, widespread pain was found to be associated with higher depression and lower self-efficacy [11]. Another study investigating psychological factors in cohorts other than whiplash found that the presence of psychological impairments were associated with the development of chronic widespread pain [51]. Psychological distress could be a consequence of widespread pain; this is supported by a randomized controlled trial that found psychological distress resolved in patients with WAD after elimination of pain [52].

In addition to psychological factors, the current literature indicates that the development of widespread pain might be associated with perceived disability and work ability. Widespread pain was shown to be significantly associated with the NDI in patients with chronic WAD [11], as well as in another chronic WAD populations, in which the same significant correlation was found between pain area and NDI [53]. Besides disability, poor work ability was associated with those who have multi-site pain (OR = 2.41) in a cohort of health care providers [54]. However, no direct measure has been conducted between work ability and widespread pain in patients with WAD. Future research may investigate such association prospectively, which might help in decision making with work capacity in WAD patients.

### 4.2. Methodological Considerations

This study has many strengths. At least 10 participants were used for each predictor variable when developing the predictive factors, which minimizes the risk of overestimating the results [55]. Moreover, allocated treatments were taken into consideration when developing the predictive factors as recommended to avoid poor performance of pain extent [56]. Finally, this study considers other potential confounders which were included in the final multivariable analysis.

There are limitations to this study that should be considered. From 205 participants, the NDI data were available from only 160 and 114 participants at one and two years, respectively; this could lead to a possible attrition bias. However, we used multivariable imputation to replace missing values with imputed values, which enables all data to be included in the final regression model. Another limitation is that the study includes patients who received a neck-specific exercise intervention with or without a behavioral approach, and this could limit the external validity of the findings when applied to other WAD populations who receive other types of exercise programs. Additionally, the final multivariable analysis may be limited by the included covariates of our analysis. Other significant covariates to pain extent and WAD outcome should be identified. Finally, the sample size of this study was slightly lower than the required number, which may have resulted in the study being underpowered. However, because this study was based on a previously published trial [22], sample size could not be increased.

## 5. Conclusions

This is the first study to evaluate the predictive ability of pain extent in individuals with chronic WAD, which, on its own, was found to be significantly associated with poor long-term outcomes. Pain extent was no longer a significant predictor once we included other predictors such as disability, psychological health, and work ability into multivariate analyses.

## Figures and Tables

**Table 1:** Baseline characteristics of the included participants with chronic WAD (*n* = 205).

Variables	Value
Sex	
Male, N (%)	72 (35%)
Female, N (%)	133 (65%)
Group	
NSE	72 (35%)
NSEB	68 (33%)
PPA	65 (32%)
Age	
Years (range) SD	40.2 (63–18) 11.5
Disability	
NDI, mean (range) SD	33.2 (76–4) 13.01
PDI, mean (range) SD	20.5 (58–0) 13.9
Pain extent	
Mean percent (range) SD	7.0 (57.3–0.00) 7.33
Neck pain intensity	
VAS, mean (range) SD	41.7 (97–0) 24.6
Quality of life	
EQ-5D, mean (range) SD *	0.6 (1–(−0.2)) 0.3
EQ-VAS, mean (range) SD **	62.8 (95–11) 18.0
Self-Efficacy	
SES, mean (range) SD	150.5 (200–47) 36.9
Fear of movement	
TSK, mean (range) SD	22.1 (41–12) 6.0
Pain catastrophizing	
PCS, mean (range) SD	18.6 (51–0) 11.2
Depression and anxiety	
HADS-A, mean (range) SD ***	6.9 (18–0) 4.3
HADS-D, mean (range) SD ****	4.8 (19–0) 4.2
Work-related factors	
WAI, mean (range) SD	35.4 (49–10) 6.9
ESES	
ESES, mean (range) SD *****	33.5 (60–6) 13.6

NSE: Neck-specific exercise; NSEB: Neck-specific exercise with a behavioral approach; PPA: Prescribed physical activity; NDI: Neck Disability Index; PDI: Pain Disability Index; VAS: Visual Analogues Scale; EQ-5D: EuroQol Five Dimension Scale; EQ-VAS: EuroQol Visual Analogue Scale; SES: Self-Efficacy Scale; TSK: 11-item Tampa Scale of Kinesiophobia; PCS: Pain Catastrophizing Scale; HADS-A and HADS-D: Hospital Anxiety and Depression Scales; WAI: Work Ability Index; ESES:.* 3; ** 4; *** 1; **** 2; ***** 1 participant’s data were missing.

**Table 2:** Selected predictor variables for response variable of NDI at 1 year.

Variables	Imputed Dataset 1	Imputed Dataset 2	Imputed Dataset 3	Imputed Dataset 4	Imputed Dataset 5
NDI	0.346	0.378	0.397	0.404	0.400
EQ5D	0	0	0	0	0
EQ-VAS	0	0	0	0	0
ESES	0	0	0	0	0
HADS-A	0	0	0	0	0
HADS-D	0.007	0.007	0.008	0.006	0.008
PCS	0	0	0	0	0
PDI	0	0	0	0	0
SES	−0.008	−0.007	−0.012	−0.008	−0.009
TSK	0	0	0	0	0
VAS	0	0	0	0	0
WAI	−0.283	−0.216	−0.260	−0.198	−0.281

NDI: Neck Disability Index; EQ-5D: EuroQol Five Dimension Scale; EQ-VAS: EuroQol Visual Analogue Scale; ESES:; HADS-A and HADS-D: Hospital Anxiety and Depression Scales; PCS: Pain Catastrophizing Scale; PDI: Pain Disability Index; SES: Self-Efficacy Scale; TSK: 11-item Tampa Scale of Kinesiophobia; VAS: Visual Analogues Scale; WAI: Work Ability Index.

**Table 3:** Selected predictor variables for response variable of NDI at 2 years.

Variables	Imputed Dataset 1	Imputed Dataset 2	Imputed Dataset 3	Imputed Dataset 4	Imputed Dataset 5
NDI	0.329	0.380	0.370	0.275	0.437
EQ5D	0	0	0	0	0
EQ-VAS	0	0	0	0	0
ESES	0	0	0	0	0
HADS-A	0	0	0	0	0
HADS-D	0	0	0	0	0
PCS	0	0	0	0	0
PDI	0	0	0	0	0
SES	0	0	0	0	0
TSK	0.090	0.085	0.041	0.036	0.072
VAS	0	0	0	0	0
WAI	−0.004	−0.002	−0.001	−0.001	−0.003

NDI: Neck Disability Index; EQ-5D: EuroQol Five Dimension Scale; EQ-VAS: EuroQol Visual Analogue Scale; ESES:; HADS-A and HADS-D: Hospital Anxiety and Depression Scales; PCS: Pain Catastrophizing Scale; PDI: Pain Disability Index; SES: Self-Efficacy Scale; TSK: 11-item Tampa Scale of Kinesiophobia; VAS: Visual Analogues Scale; WAI: Work Ability Index.

**Table 4:** Crude association between baseline pain extent to 1 year NDI outcome.

	β	SE	T Value	df	*p* Value	Low 95%CI	Upper 95% CI	Adjusted R^2^
(Intercept)	24.004	1.712	14.02	106.709	0	20.61	27.398	0.05
Pain Extent	0.534	0.186	2.88	39.717	0.006	0.159	0.909	

β: Unstandardized Coefficient; SE: Standard Error; CI: Confidence Intervals; Adjusted R^2^: represents the variance in NDI as explained by the variable.

**Table 5:** Adjusted associations between baseline pain extent to 1 year NDI outcome.

	β	SE	T-Value	df	*p* Value	Low 95%CI	Upper 95% CI	Adj R^2^
(Intercept)	24.516	15.147	1.618	25.561	0.118	−6.646	55.678	0.31
Pain Extent	0.11	0.187	0.586	21.34	0.564	−0.28	0.499	
NSEB Group	1.319	3.059	0.431	23.442	0.67	−5.003	7.64	
PPA Group	7.82	2.857	2.737	38.859	0.009	2.04	13.6	
HADS-D	0.302	0.324	0.933	143.995	0.353	−0.339	0.943	
NDI	0.401	0.143	2.809	29.079	0.009	0.109	0.693	
SES	−0.023	0.046	−0.509	42.047	0.613	−0.115	0.069	
WAI	−0.332	0.266	−1.249	31.169	0.221	−0.873	0.21	

NSEB: Neck-specific exercise with a behavioral approach; PPA: Prescribed physical activity; HADS-D: Hospital Anxiety and Depression Scales; NDI: Neck Disability Index; SES: Self-Efficacy Scale; WAI: Work Ability Index; β: Unstandardized Coefficient; SE: Standard Error; CI: Confidence Intervals; Adjusted R^2^: represents the variance in NDI as explained by the variable.

**Table 6:** Crude association between baseline pain extent to 2 years NDI outcome.

Predictor	β	SE	T Value	df	*p* Value	Low 95%CI	Upper 95% CI	Adjusted R^2^
(Intercept)	24.916	1.926	12.937	25.715	0	20.955	28.877	0.04
Pain Extent	0.485	0.204	2.383	17.731	0.029	0.057	0.914	

β: Unstandardized Coefficient; SE: Standard Error; CI: Confidence Intervals; Adjusted R^2^: represents the variance in NDI as explained by the variable.

**Table 7:** Adjusted associations between baseline pain extent to 2 years NDI outcome.

Predictors	β	SE	T Value	df	*p* Value	Low 95%CI	Upper 95% CI	Adjusted R^2^
(Intercept)	7.462	16.296	0.458	11.963	0.655	−28.056	42.981	0.25
Pain Extent	0.159	0.186	0.856	22.178	0.401	−0.226	0.544	
NSEB Group	2.154	3.818	0.564	9.858	0.585	−6.37	10.678	
PPA Group	8.518	3.899	2.185	9.513	0.055	−0.229	17.266	
NDI	0.387	0.178	2.173	9.493	0.056	−0.013	0.787	
TSK	0.3	0.265	1.132	14.927	0.276	−0.266	0.866	
WAI	−0.089	0.331	−0.269	9.554	0.793	−0.832	0.654	

NSEB: Neck-specific exercise with a behavioral approach; PPA: Prescribed physical activity; NDI: Neck Disability Index; TSK: 11-item Tampa Scale of Kinesiophobia; WAI: Work Ability Index; β: Unstandardized Coefficient; SE: Standard Error; CI: Confidence Intervals; Adjusted R^2^: represents the variance in NDI, as explained by the variable.

## Data Availability

Raw data that support the findings of this study are available from the corresponding author, upon reasonable request.
